# Mechanical and Water Absorption Properties of Waterborne Polyurethane/Graphene Oxide Composites

**DOI:** 10.3390/ma16010178

**Published:** 2022-12-25

**Authors:** Sergey A. Baskakov, Yulia V. Baskakova, Elizaveta V. Dvoretskaya, Svetlana S. Krasnikova, Valentina A. Lesnichaya, Yury M. Shulga, Gennady L. Gutsev

**Affiliations:** 1 Federal Research Center of Problem of Chemical Physics and Medicinal Chemistry, Russian Academy of Sciences, 142432 Moscow, Russia; 2 Department of Functional Polymer Materials, National University of Science and Technology MISiS, Leninsky pr. 4, 119049 Moscow, Russia; 3 Department of Physics, Florida A&M University, Tallahassee, FL 32307, USA

**Keywords:** waterborne polyurethane, graphene oxide, composite, mechanical properties, water absorption

## Abstract

Nanocomposites based on waterborne polyurethane (WPU) and graphene oxide (GO) have been synthesized and characterized. It was found that after the incorporation of GO, WPU films became mechanically more rigid, and the Young’s modulus increased by almost six times. It is shown that the lateral size of GO sheets influences the mechanical properties of WPU/GO composites. In particular, composites with larger lateral size of GO sheets have higher values of Young’s modulus. Additionally, if the mechanical properties are improved with the addition of GO additive, then water absorption decreases for WPU modified with small GO sheets whereas it increases for WPU modified with large GO sheets. Possible reasons for this behavior are discussed.

## 1. Introduction

Quite recently, the use of graphene materials (graphene nanoplates, graphene oxide (GO), reduced graphene oxide (RGO), etc.) for the preparation of polymer composites opened a new attractive area in materials science [1,2,3,4,5,6]. Graphene-containing polymer composites demonstrated a significant improvement in mechanical and physicochemical properties as well as operational characteristics with respect to those of the initial polymers. Polyurethanes (PU) are widely used for the manufacture of sealing products, cleaning abrasive-resistant surfaces, elastic forms, decorative elements, paints and varnishes, adhesives, sealants, machine components and machine parts (shafts, rollers, belts, etc.), various rubber-technical products for domestic and industrial purposes [7]. Among other polymeric materials, PU is distinguished by high mechanical strength, large elasticity range, high abrasion resistance, and excellent impact-viscosity properties. In addition, it is relatively easy to control the properties of PU by changing the types and amounts of isocyanate, polyol, surfactants, and fillers [8,9]. Many properties of conventional PU can be significantly improved by adding graphene oxide as a nanomodifier.

The main task in the development of composites with improved characteristics is obtaining the maximum dispersion of nanofillers in the polymer matrix. High dispersion of nanoparticles makes it possible to achieve a significant improvement in properties of a composite at a low filler content in the polymer. Phase incompatibility and/or insufficient exfoliation of graphene before mixing with the polymer can lead to the formation of large agglomerates. To prevent agglomeration, one can introduce a graphene modifier into PU after its mixing with one of the PU components before polymerization. Monomers of PU are usually aliphatic or aromatic diisocyanates (DI), which are very sensitive to moisture and possess a high viscosity; therefore, the introduction of GO into DI or its derivatives can adversely affect the composite properties if GO dispersion is insufficient and moisture ingress is not sufficiently prevented. The second component is a polyol, which is a polymerization product of ethylene oxides with a small molecular weight (up to 0.6 kDa). Polyols have a stable viscosity and are more suitable for the introduction of graphene materials [10]. Sometimes GO is introduced into a mixture of two components in the form of a powder [11] or a solution [12].

In recent years, polyurethane manufacturers have shifted their focus towards the production of specialized and environmentally friendly polyurethanes of high quality. Among them, waterborne polyurethane (WPU) has attracted the attention of researchers and industry representatives because of its absence of odor, convenient storage, and safety of use [13,14,15,16]. In addition to environmental benefits, WPUs combine other useful properties such as a high PU value in emulsion, high molecular weight, and acceptable viscosity. In addition, they also have low temperatures of the film formation, which is important when WPUs are used as a base for paints, varnishes, sealants, etc. [17]. However, WPU also has certain disadvantages such as slow drying speed and high cost compared to PU solutions as well as high pH sensitivity. To expand the scope of WPU applications, it is necessary to improve its complex characteristics by means of appropriate modifications. An analysis of literature sources has shown that many researchers have paid more attention to the manufacture and certification of WPU and WPU/GM composite materials (GM is a graphene material) [13,14,15,16].

However, the processes of WPU modification with graphene materials have not yet been fully understood because of structural diversity of both matrix material (WPU) and GM. There is insufficient information in the literature on the water absorption of WPU modified with pure GO. Graphene oxide has hydroxyl (−OH), carboxyl (−COOH), carbonyl (−C=O) and epoxy (C−O−C) groups on its surface and is a hydrophilic material. Therefore, the degree of GO swelling (see, for example, [11]) may be increased when improving mechanical properties of WPU by GO doping, which is undesirable. To prevent GO swelling in the process of a WPU modification, one can use either reduced GO or GO pre-modified in such a way as to reduce the water absorption by the composite. For example, GO was modified using 3-aminopropyltriethoxysilane (APTES) and (3-glycidyloxypropyl)triethoxysilane (GPTS) [18,19]. It was also reported [20,21] that 4,4′-methylenebis(phenylisocyanate) (MDI) and 6-(bis(2-hydroxyethyl)amino)dibenzo[c,e] [1,2]oxaphosphinine 6-oxide (DEA-DOPO) was used to functionalize GO. Several research groups also used reduced graphene oxide [22,23,24,25,26]. Studies of WPU/GO composites, where graphene oxide has not been modified have also been performed [11,19,27,28,29,30]. 

It was found [27] that small additions of GO (0.01–0.10 wt%) can change the adhesion properties of WPU films. It was noted [28] that the thermal stability and mechanical properties of the PU/GO composite can be significantly improved if modified GO is used instead of pure GO. According to [29], the breaking tension of WPU/GO films depends on the GO content and passes through a maximum. It was also shown [28,30] that pre-modified GO can be a more effective additive in a WPU/GO composite compared to pure GO. Two series of PU/GO composites, which differed in the type of isocyanates (methylene diisocyanate (MDI) and hexamethylene diisocyanate (HMDI) and where GO was introduced in different amounts from 0.5% to 2.0%, were synthesized in Ref. [11]. The water absorption in both series ranged from 10% to 30% increasing with an increase in the GO content. Samples of the second series exhibited a stronger tendency toward water absorption compared to the samples of the first series. On the contrary, it was found [19] that the introduction of 0.2% GO into WPU reduces its swelling degree from 52.34% to 8.59%. 

This apparent contradiction will be resolved in the present work, in which we synthesize two series of WPU/GO mixtures with the GO content in the range from 0.1% to 2.0 wt%. These two series differed in the lateral size of graphene oxide particles and were used to obtain WPU/GO composite films. The resulting films were certified by elemental analysis, thermogravimetry, and IR spectroscopy. The main purpose of this work is to study the effect of GO additives on the mechanical and water-absorbing properties of the samples obtained.

## 2. Experimental

### 2.1. Materials 

The aliphatic anionic polyurethane dispersion Bayhydrol^®^ UH 340/1 (COVESTRO, Leverkusen, Germany) was used as a source of polyurethane. This material is used as a binder in the formulation of highly elastic protective and decorative coatings for wood, metal, and plastic, as well as a combined binder to improve the elasticity and flexibility of such coatings. Graphite oxide was obtained by the modified Hummers method [31] according to the procedure described in [32]. Two fractions of low-ash graphite with different flake sizes: 30 µm or less (the first fraction) and 210 µm or less (the second fraction) were used as raw materials. A GO suspension of a given concentration was prepared by ultrasonic treatment of a graphite oxide suspension followed by centrifugation at 3000 g to remove large nonsegregated particles.

### 2.2. Film Preparation 

Composite films of WPU/GO were prepared as follows. The calculated volume of the GO suspension was added dropwise to the WPU dispersion with stirring which continued for 10 min after the process of introduction of GO was over. Next, the mixture was poured into a mold made of a glass plate edged with the sidewall to prevent spreading the mixture. The mold was pre-aligned to the bar level for obtaining a film without significant fluctuations of its thickness. After the mixture had dried, the film was removed from the mold and was used in subsequent experiments.

### 2.3. Equipment 

Elemental analysis of the GO samples was carried out on a Vario Micro cube CHNS analyzer (Elementar GmbH, Hanau, Germany).

Thermogravimetric analysis (TG) of the samples was performed using an STA 449 F3 Jupiter instrument (Selb, Bavaria, Germany). To calibrate the balance, air in the instrument chamber was pumped out to (10^−2^ bar) and the chamber was filled with He gas of grade 6.0 (99.9999%). After that, two empty corundum (Al_2_O_3_) crucibles were mounted on the holder in the chamber and the baseline was recorded. Then, a sample was placed in one of the empty crucibles, and the chamber was pumped out and again filled with helium. The measurements were carried out in the temperature range of 20–400 °C at a rate of 10 °C/min in a He flow of 50 mL/min. 

The IR spectra were recorded at room temperature in the range of 400–4000 cm^−1^ on a Perkin-Elmer “Spectrum Two” Fourier-transform spectrometer (Waltham, Massachusetts, United States) equipped with an ATR attachment with a diamond crystal.

Stress (σ) − strain (ε) curves were obtained on a Zwick/Roell Z010 («Zwick GmbH & Co. KG», Ulm, Germany) universal testing machine in accordance with ASTM 882 Standard Test Method for Tensile Properties of Thin Plastic Sheeting. All samples had a rectangular shape with sizes of 100 mm × 10 mm. The length between two grips was 50 mm. Elongations at break (εb), maximum tensile stresses (σmax) and standard deviations for these values were estimated from data obtained for 10 test samples of each film.

Optical micrographs of graphene oxide particles were obtained by using an Olimpus BX43 optical microscope (Olympus Corp., Tokyo, Japan) in a bright field and using phase contrast and magnification from 200× to 400×.

### 2.4. On Swelling Study 

For an estimation of the swelling degree, three samples of size 5 × 1 × 0.1 cm were made for each composition. Before testing, the sample surfaces were cleaned using isopropyl alcohol, dried in air for 2 h at a temperature of 23 °C and a humidity of 50%, and then in an oven for 24 h at a temperature of 50 °C. After drying, the samples were cooled to a temperature of 23 ± 2 °C, weighed and placed in a vessel with distilled water at room temperature (23 °C) for 2 h. Before weighing, the surface of each sample removed from the water was dried using dry filter paper for no more than one minute. The mass fraction of water absorbed by the sample (or swelling degree) was calculated by the formula

SwD = [(m_w_ − m_0_)/m_0_] × 100 (1)

where m_w_ is the mass of the test sample after soaking in water, m_0_ is the mass of the test sample before immersion in water (see Ref. [33]). The test result was taken as the arithmetic mean of the three values obtained with the same duration of soaking in water.

## 3. Results and discussion 

### 3.1. Optical Photographs

**Figure 1:**
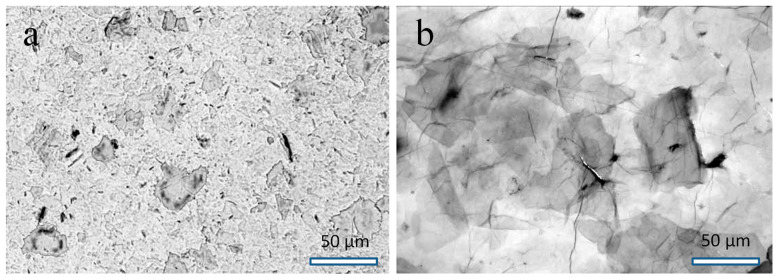
Optical micrographs of GO1 (**a**) and GO2 (**b**) samples.

To assess the influence of GO particle sizes on the properties of WPU/GO films, we used GO obtained from graphite of grades GSM−2 and GK−1, which differ in the average size of ingots. Figure 1 shows micrographs illustrating differences in the lateral sizes of GO particles obtained from graphite of different grades. As could be seen, the GO particle size correlates with the size of the initial graphite flakes. We denote samples obtained from fine-grained graphite GK−1 and coarse-grained graphite GSM−2 as GO1 and GO2, respectively.

### 3.2. Elemental Analysis

The elemental compositions of GO samples studied are shown in Table 1. One can notice that all samples are similar with respect to the content of C, O, and H (the differences are less than 1 wt%). It should be noted that the sulfur content in the GO1 sample is noticeably higher than that of the GO2 sample. This is because GO1 has smaller particles, which makes it difficult to clean them from sulfuric acid residues by centrifugation. It can also be seen that both samples do not contain nitrogen. Contents some WPU/GO1 samples are close to that for WPU. 

### 3.3. IR Spectroscopy

**Figure 2:**
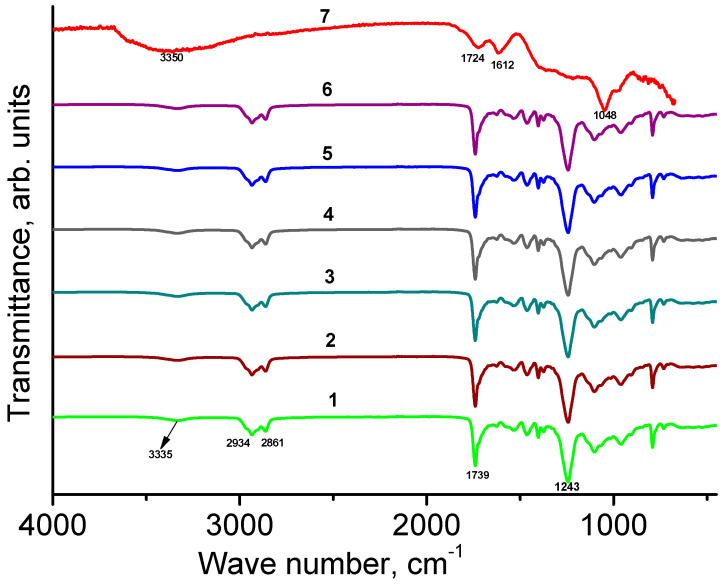
IR spectra of WPU film (**1**), WPU/GO1 composites with GO1 content of 0.1 (**2**), 0.5 (**3**), 1.0 (**4**), 1.5 (**5**), and 2.0 (**6**) wt%, and GO1 powder (**7**).

The IR spectra of GO1, WPU film, and WPU/GO1 composites are shown in Figure 2. The IR spectrum of GO1 (Figure 2, curve 7) contains a number of overlapping absorption bands in the range of 3700–3000 cm^−1^ are which can be attributed to the stretching vibrations of the O−H bonds of groups and water molecules [34,35,36]. An absorption band at 1724 cm^−1^ is due to C−O vibrations in carbonyl groups and/or ketones. Both vibrations of C=C double bonds [37] and the bending vibrations of water molecules contribute to the band intensity at 1612 cm^–1^. Absorbtion band at 1048 cm^−1^ is due to vibrations of C−O−C bonds of epoxy groups [38].

The absorption band in the spectrum of WPU film at ~3335 cm^–1^ is due to stretching vibrations of N−H bonds of the urethane block [22,28,30,39,40]. Bands at 2934 cm^−1^ and 2861 cm^−1^ correspond to stretching vibrations of C−H bonds. Vibrations of C=O bonds correspond to the band at 1739 cm^−1^. An assignment of the intense peak at 1243 cm^−1^ in the literature is ambiguous. In accordance with [41], we relate this band to ν(C−O) of the urethane group. The absence of an isocyanate band at 2275 cm^−1^ indicates that the initial polymer is free of unreacted monomers since −N=C=O groups from MDI were successfully linked to the formation of urethane bonds [42].

All absorption bands of the unmodified polyurethane matrix occur in the IR spectra of modified WPU/GO nanocomposites as well. We did not find any significant influence of GO addition on the IR spectra of PU composites and no noticeable shifts of the absorption bands were observed. No significant difference between the IR spectra of PU and PU/GO were found by the authors of a recent study [43] as well. The low sensitivity of the IR spectra to the addition of GO could be associated with low concentrations of GO and the high polarizability of local dipoles of the polymers.

### 3.4. Thermogravimetric Analysis

**Figure 3:**
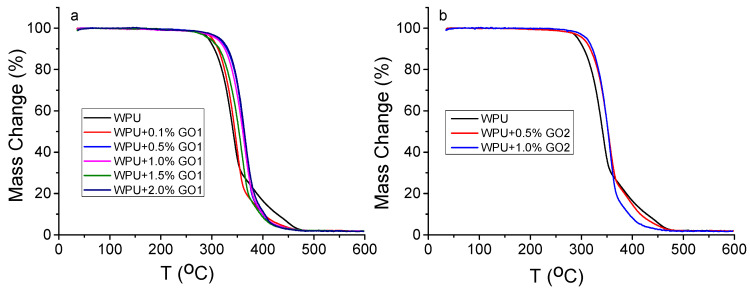
TG curves for WPU/GO1 (**a**) and WPU/GO2 (**b**) samples.

TGA curves of WPU/GO1 and WPU/GO2 samples are shown in Figure 3. As could be seen, the same trend in mass loss is observed for all samples. A slight mass loss at 100°C is due to the loss of water, low molecular weight organic solvents, and impurities. On the TGA curves of WPU, there are two sections in the range from 280 °C to 460 °C with the highest mass loss. These curve sections are related with thermal degradation of the soft segment (polyol chains) in the range of 280–350 °C and decomposition of the hard segment of WPU (urethane groups NHCO) in the range of 350−460 °C. Thus, thermal degradation of WPU and WPU/GO composites occurs in two stages, which correspond to the thermodynamic incompatibility of two segments of the WPU matrix [43,44].

The introduction of graphene oxide into the WPU polymer matrix leads to an increase in thermal stability of the polymer. This can be explained by the presence of a large number of oxygen-containing groups, which play an effective role in the character of chemical reactions and intermolecular interactions with the matrix. GO forms spatial bonds with the polymer framework, which restrict the movement of polyurethane molecular chains.

On the TG curves of GO1 and GO2 composites, there is a shortening of the curve parts responsible for the destruction of rigid segments of PU molecules. The values of the initial decomposition temperature at a 5 wt% mass loss depend on the GO content in the composites and increase from 293 °C to 320 °C for WPU/GO1 with 2.0 wt% of GO to 313 °C for WPU/GO2 with 2.0 wt% of GO. One can state that the maximum value of the initial decomposition temperature is achieved with the addition of GO with smaller lateral particle sizes.

### 3.5. Mechanical Properties

**Figure 4:**
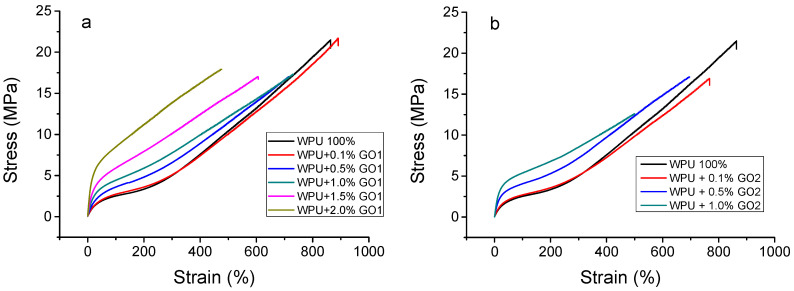
Stress–strain dependence for WPU/GO1 (**a**) and WPU/GO2 (**b**) samples.

Mechanical properties of WPU/GO with different GO content were investigated by means of a tensile test. According to the test results presented in Figure 4 and Table 2, there is a significant increase in the elastic modulus of PU films due to adding GO of both types, whereas the tensile strength of composite films tends to decrease. GO addition generally leads to a reduced relative elongation to disruption of all composites, and the higher GO content in the composite, the lower the relative elongation. The WPU film with 2 wt% of OG1 has the highest average Young’s modulus of 42.95 MPa among all studied WPU/OG1 composites, which corresponds to an increase of 569% compared to the Young’s modulus of WPU (7.55 MPa).

Mechanical test data show a significant effect of GO particle sizes on the mechanical properties of the composites. Thus, the Young’s modulus for WPU with 1 wt% GO1 is 18.23 MPa, and it increases to 30.15 MPa for samples containing 1 wt% GO2. A similar dependence of the Young’s modulus is observed for all samples with the same concentrations of GO1 and GO2. The stress at the moment of specimen break increases from 18.94 to 21.63 MPa after the addition of 0.1% wt GO1. In all other cases, the reduction of this index is 5–15%.

Graphene-like fillers have been intensively used to improve the mechanical properties of polyurethanes [11,45,46,47,48,49,50,51,52,53,54,55,56]. These nanomodifiers, when added in small amounts (up to 4%), generally lead to a significant improvement in the properties of the polyurethane matrix due to the interaction between polyurethane chains and fillers at the nanoscale. According to our understanding of the literature data, mechanical hardening occurs mainly as a result of physical crosslinking between the rigid domains of the PU matrix and functional groups on the GO surface through the formation of hydrogen bonds. Moreover, some authors admit (see, for example, [57]) that the hydroxyl functional groups of GO are well suited for the formation of composites with polyurethane via chemical bonding.

### 3.6. Water Absorption

Our study of water absorption, whose results are presented in Table 3, showed that the absorption increases for the PU/GO2 composite compared to that of the original PU, whereas it decreases for the PU/GO1 composite. Interestingly, the dependence of water absorption on GO concentration in the PU/GO2 composite passes through a minimum, which is reached at a concentration of 1 wt%. Such a difference between composites with GO1 and GO2 could be due to the fact that the lateral size of GO2 sheets are 10 times larger than the lateral size of GO sheets. It is possible that the presence of larger sheets leads to the formation of additional channels or pores for moisture to penetrate into the material. Another possible reason for this difference in water absorption between the composites may be the different concentration of hydroxyl groups on the surface of GO1 and GO2.

## 4. Conclusions

It was demonstrated that the introduction of a small additive of GO (up to 2 wt%) effectively strengthens the PU matrix, while the value of the Young’s modulus grows symbatically with increasing concentration of the additive. The WPU film with 2 wt% of GO has the highest average Young’s modulus of 42.95 MPa, which corresponds to an increase of 569% compared to the Young’s modulus of initial WPU (7.55 MPa). Mechanical test data show a significant effect of GO particle sizes on the mechanical properties of the composites. Thus, the Young’s modulus for WPU with 1 wt% GO1 (lateral size of sheet 30 µm or less) is 18.23 MPa, and it increases to 30.15 MPa for samples containing 1 wt% GO2 (lateral size of sheet 210 µm or less). In accordance with the literature data, this behavior occurs due to physical crosslinking between the rigid domains of the PU matrix and functional groups on the GO surface through the formation of hydrogen bonds.

An important parameter for paintwork and waterproofing materials is their water absorption. The measurements of water absorption showed that water absorption decreases for polyurethane modified with small-size GO whereas it increases for polyurethane modified with large-size GO. Graphene oxide, as is known (see, for example, [32]), depending on the degree of reduction, can be both hydrophilic and hydrophobic. It is possible that not only the degree of reduction, but also the GO sheet conformation can affect the water absorption properties of both GO and VPU/GO composites. Such studies may be the subject of our future work. 

## Figures and Tables

**Table 1:** Chemical composition (in wt%) of the graphene oxides, WPU, and some WPU/GO1 samples. (Oxygen content was estimated by the formula [O] = 100 − $\sum_{i}[C_{i}$], where [*C_i_*] is the content of the *i*-th element.)

Sample	C	O	H	N	S
GO1	45.28	49.70	2.72	0.00	2.30
GO2	45.32	50.82	3.03	0.00	0.83
WPU	58.03	29.96	8.74	2.99	0.29
WPU/GO1 (0.5 wt%)	57.76	30.18	8.74	2.96	0.37
WPU/GO1 (1.0 wt%)	58.14	29.55	8.84	3.07	0.41
WPU/GO1 (1.5 wt%)	57.89	30.21	8.77	2.94	0.19

**Table 2:** Average numerical data of mechanical tests for WPU films and WPU/GO composites.

GO Content	Young’s Modulus, MPa		* σ_b_, MPa		** ε_b_, %	
	WPU/GO1	WPU/GO2	WPU/GO1	WPU/GO2	WPU/GO1	WPU/GO2
0%	7.55	7.55	18.94	18.94	790	790
0.1%	8.69	9.90	21.63	17.44	894	772
0.5%	11.96	18.52	15.66	16.07	678	680
1%	18.23	30.15	16.65	12.05	700	501
1.5%	25.42	-	16.66	-	640	-
2%	42.95	-	15.81	-	433	-

* **σ_b_** = stress at the moment of specimen break; ** **ε_b_** = elongation at break.

**Table 3:** Water absorption of the samples studied (in wt%, see Equation (1)).

Sample	SwD for WPU/GO1	SwD for WPU/GO2
Initial WPU	27.52 ± 0.99	27.52 ± 0.99
0.1% GO	25.69 ± 2.04	32.95 ± 1.16
0.5% GO	23.84 ± 1.01	33.94 ± 2.31
1% GO	21.91 ± 0.67	30.76 ± 0.91
1.5% GO	23.19 ± 1.15	-
2% GO	27.45 ± 1.38	-
